# Sedative Agents, Synthetic Torpor, and Long-Haul Space Travel—A Systematic Review

**DOI:** 10.3390/life15050706

**Published:** 2025-04-27

**Authors:** Thomas Cahill, Nataliya Matveychuk, Elena Hardiman, Howard Rosner, Deacon Farrell, Gary Hardiman

**Affiliations:** 1 Faculty of Medicine, Health and Life Sciences, School of Biological Sciences, and Institute for Global Food Security, Queen’s University Belfast, Belfast BT9 7BL, Northern Ireland, UK; tcahill01@qub.ac.uk (T.C.); nmatveychuk01@qub.ac.uk (N.M.); 2 St Luke’s Campus, University of Exeter Medical School, Exeter B3183, UK; eh805@exeter.ac.uk; 3 Department of Anesthesiology, Cedars Sinai Medical Center, Beverly Hills, CA 90048, USA; howard.rosner@cshs.org (H.R.); deaconfarrellmd@gmail.com (D.F.); 4 Department of Medicine, Medical University of South Carolina (MUSC), Charleston, SC 29425, USA

**Keywords:** induced torpor, sedative agents, long-duration space missions, synthetic torpor, space exploration, inhalation anesthetics, metabolic reduction, microgravity, spaceflight countermeasures, torpor induction and maintenance, pharmacokinetics, pharmacodynamics, spaceflight physiology, radiation protection, extended anesthesia applications

## Abstract

Background: With renewed interest in long-duration space missions, there is growing exploration into synthetic torpor as a countermeasure to mitigate physiological stressors. Sedative agents, particularly those used in clinical anesthesia, have been proposed to replicate aspects of natural torpor, including reduced metabolic rate, core temperature, and brain activity. Objectives: This systematic review aims to evaluate the potential of sedative agents to induce torpor-like states suitable for extended spaceflight. The review specifically investigates their pharmacokinetics, pharmacodynamics, and performance under space-related stressors such as microgravity and ionizing radiation. Methods: We conducted a comprehensive search across multiple databases (e.g., PubMed, Scopus, Web of Science) for studies published from 1952 to 2024. Eligible studies included experimental, preclinical, and clinical investigations examining sedative agents (especially inhalation anesthetics) in the context of metabolic suppression or space-relevant conditions. Screening, selection, and data extraction followed PRISMA guidelines. Results: Out of the screened records, 141 studies met the inclusion criteria. These were thematically grouped into seven categories, including torpor physiology, anesthetic uptake, metabolism, and inhalation anesthetics. Sedative agents showed variable success in inducing torpor-like states, with inhalation anesthetics demonstrating promising metabolic effects. However, concerns remain regarding delivery methods, safety, rewarming, and the unknown effects of prolonged use in space environments. Conclusions: Sedative agents, particularly volatile anesthetics, hold potential as tools for inducing synthetic torpor in space. Nevertheless, significant knowledge gaps and technical challenges persist. Further targeted research is required to optimize these agents for safe, controlled use in spaceflight settings.

## 1. Introduction

### 1.1. The Rise in Space Exploration and Its Challenges

The 20th century realized the beginning of space-related missions, originally a manifestation of the geopolitical competition to be the first to send a human to the moon [1]. The space race quickly evolved into international cooperation for scientific research fueled by the prospect of economic returns, and by 2011, there were over 60 active space missions, with data showing a year-on-year increase in payloads sent into orbit [2,3]. Today, this international cooperation between commercial and international partners has culminated in NASA’s Artemis Program, which aims to develop a lunar base and innovative technologies that will set the foundations for a human mission to Mars [4]. This would entail a 7-month transit from Earth to the red planet, a year-long stay, and a 7-month return journey, considering favorable orbital alignments. It is with this earth-independent interplanetary travel ambition that a whole new set of challenges arises. To elaborate, a journey of this length will require advancements in the space food system, particularly long shelf-life foods with high nutritional value. Interplanetary travel will also require effective strategies to combat the increased exposure to microgravity and galactic cosmic rays when traveling beyond Earth’s gravitational field and magnetic shield. High-energy galactic cosmic rays pose a serious threat to the integrity of cellular DNA in astronauts [5] as they can cause double-strand breaks, increasing the chance of cell death, mutagenesis, or promoting the development of cancer [6]. Additionally, prolonged exposure to microgravity increases bone resorption [7] and causes skeletal muscle atrophy [8]. Considerable research has been carried out to evaluate effective countermeasures ranging from anti-oxidant supplementation to counter reactive oxygen species, shielding materials against radiation [9], or resistive exercise routines to counteract muscle atrophy from disuse in microgravity [10,11]. An induced torpor-like state has emerged as a single countermeasure that might mitigate against many of these space-related health challenges, and it has been the source of much discussion in the scientific community [12].

### 1.2. Systematic Review

The objective of this systematic review was to comprehensively explore and synthesize the existing research on the physiological, technological, and medical adaptations required for long-duration space exploration. Specifically, it aimed to do the following:

Investigate advancements in bioastronautics, focusing on human performance optimization and medical innovations to support astronauts in extreme environments.

Examine the potential of hibernation and metabolic modulation as strategies for reducing metabolic demands and improving safety during extended space missions.

Analyze the applications of therapeutic hypothermia and anesthesia in space, particularly their relevance to critical care and long-duration missions.

## 2. Methodology

This systematic review was conducted following the PRISMA (Preferred Reporting Items for Systematic Reviews and Meta-Analyses) guidelines to ensure transparency and rigor. The process involved four key phases: identification, screening, eligibility, and inclusion.

### 2.1. Identification

A comprehensive search strategy was developed to capture all of the relevant literature over the past two decades. The search was conducted in multiple electronic databases, including PubMed, Scopus, Web of Science, and IEEE Xplore. Search terms and combinations included keywords such as “space exploration”, “bioastronautics”, “hibernation”, “therapeutic hypothermia”, “radiation protection”, “metabolic adaptation”, and “anesthesia in space”. Additional records were identified through manual searches of reference lists and conference proceedings. The search yielded 2562 total records. All retrieved records were exported into reference management software, and duplicate entries were removed.

### 2.2. Screening

After duplicates were removed, 1357 unique records were screened based on titles and abstracts. The inclusion criteria at this stage required articles to address: physiological or technological adaptations for space exploration, applications of hibernation, metabolic modulation, or radiation protection; or anesthesia or hypothermia methods with relevance to long-duration space missions. Exclusion criteria included studies without original data, opinion pieces, and research focused solely on terrestrial applications unrelated to space exploration. A total of 88 records were excluded during this phase.

### 2.3. Eligibility

The remaining full-text articles were assessed for eligibility using a detailed set of inclusion criteria: peer-reviewed studies, systematic reviews or primary research studies with detailed methodologies, studies providing quantitative or qualitative data on space mission adaptations. Articles were excluded if they lacked sufficient data, did not meet methodological quality standards, or did not address the review’s objectives.

### 2.4. Inclusion

**Figure 1:**
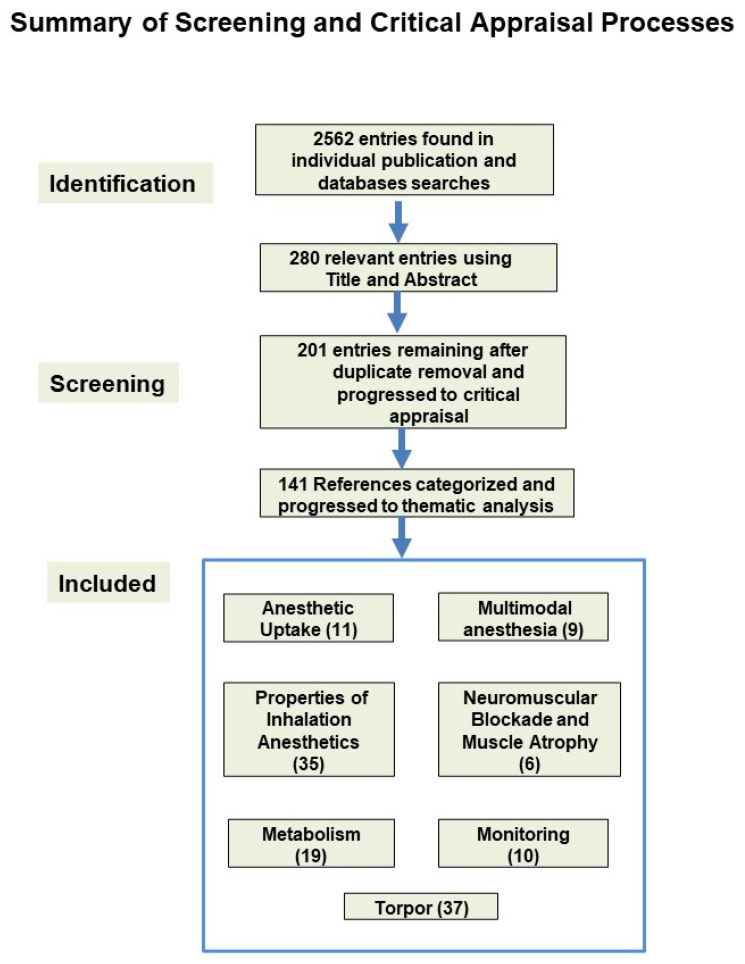
Summary of screening and critical appraisal processes.

A total of 141 studies were included in the systematic review. These studies were then categorized into seven primary themes: torpor (and induced torpor), anesthetics uptake, metabolism, multimodal anesthesia, properties of inhalation anesthetics, monitoring, neuromuscular blockade and muscle atrophy and metabolism (Figure 1). Data was extracted from the included studies using a standardized form that captured the study objectives, methods, key findings, and relevance to space exploration. The results were synthesized qualitatively, with emphasis on identifying trends, gaps, and implications for future research (Supplemental Tables S1 and S2).

### 2.5. Methodological Framework for Systematic Review: Assessing Bias, Effectiveness, and Evidence Certainty

This review encompasses highly diverse fields—space biology, torpor, and anesthesia—each contributing unique insights to address the physiological and technological challenges of long-duration space exploration. The AMSTAR 2 (A Measurement Tool to Assess Systematic Reviews 2) checklist was applied. Each study was classified as having a low, moderate, or high risk of bias, and results were used to inform the synthesis process. To minimize the risk of bias in this review, a wide range of articles was utilized as sources. Through their analysis, it allowed us to identify recurring information as well as gaps in the literature. When information was presented in only a single article, efforts were made to locate additional scientific works on similar topics to explain discrepancies and validate the findings. The credibility of the selected sources was established using reputable databases, which are widely recognized for their reliability and have undergone consistent verification. Additionally, the authors of the chosen studies possess expertise in their respective fields, demonstrated by their academic qualifications and professional achievements, further supporting the trustworthiness of the sources.

## 3. Torpor

A total of 141 studies were included in the systematic review and were grouped into seven overarching themes: torpor and induced torpor, anesthetic uptake, metabolism, multimodal anesthesia, properties of inhalation anesthetics, monitoring, and neuromuscular blockade with associated muscle atrophy and metabolism (Figure 1).

Torpor, in its natural form, is a physiological state characterized by a reduction in body temperature and metabolism. This is often accompanied by physiological changes such as a reduction in respiration rate, heart rate, and reduced regional brain activity in the neocortex and the reticular formation, followed by thalamocortical structures and the limbic system, as measured by electroencephalogram (EEG) and implanted electrodes [13,14,15]. It is exploited by heterotherms as a mechanism to conserve energy during times of food scarcity and/or low temperatures to increase survival rates [16]. It has been estimated that around 30–50% of the ~5500 species of mammals worldwide are heterothermic, roughly 10% of the ~10,000 avian species [17], not including the metabolic depression observed during torpor, dormancy, and estivation seen in gastropods, fish, and reptile species [18]. Each species displays a unique phenotype in thermo-regulation and metabolic suppression [19] with smaller mammals tending to rely heavily on body temperature reduction to reduce metabolic rate.

For instance, torpor utilized by the arctic ground squirrel exploits a body temperature as low as −2.9 °C with reductions in metabolism of >99% in relation to resting metabolism [14]. These reductions facilitate and/or are facilitated by a reduction in heart rate from 200–300 beats per minute (bmp) to 3–4 bpm with a significant drop in blood flow and respiration [20]. In contrast, the hibernating black bear undergoes a drop in core body temperature from 36 °C down to 30 °C, enabling a reduction in heart rate from 55 to as few as 9 bpm, a reduction in respiration from 6–10 breaths per minute to 1 breath every 45 s, with a physiological inhibition of its metabolism to approximately 25% of basal rates; below that which would be expected from reduced temperatures alone [21,22,23]. In comparison with the arctic ground squirrel, whose torpor is interrupted by inter-bout arousals used to remove harmful waste products [24], bears can spend up to 7 months in torpor where they do not eat, drink, urinate or defecate, utilizing energy stored as white adipose tissue [23]. These slight decreases in core body temperature are generally reversible and carry a lower risk of the severe complications associated with extreme hypothermia [25,26]. Additionally, bears are of a more comparable basal metabolic rate and weight to humans than rodents and it is therefore generally accepted that this model of torpor may be the most practical and achievable in humans [27,28].

### 3.1. Induced Torpor as a Countermeasure for the Challenges of Spaceflight

The challenges of long-term space travel, such as microgravity, radiation exposure and metabolic stress necessitate innovative countermeasures. An induced torpor-like state in astronauts might alleviate the negative consequences of long-term space travel and make interplanetary travel safer and more accessible. This has been recognized by world’s leaders in the space industry with both NASA [29] and ESA [27] reporting on the benefits and viability of an induced torpor-like state utilization for crew during space travel. The efficacy of an induced torpor-like state as a countermeasure for radiation has been evaluated in studies of induced hypothermia using both in vitro [30] and in vivo [31,32,33] models, which have both demonstrated radio-protective effects: reviewed further by Cerri, M., et al. [34].

In vitro experiments using cell lines have demonstrated the potential for the radioprotective effects of hypothermic conditions. For instance, research using human lymphoblastoid TK6 cells has shown that pre-exposing the cells to 0.8 °C prior to exposure to 1Gy of γ- or X-rays results in radioprotective effects, postulated to be a result of increased DNA double strand repair mechanisms through a shift in the cell cycle [35].

Additionally, animal models have revealed regulatory mechanisms behind the radioprotective effects of hypothermic conditions. For example, one study utilized the poikilothermic nature of zebrafish to examine the effects of lowered body temperatures on radioprotective. A hypothermic state was achieved in zebrafish using melatonin (24 mM) as a sedative and a reduction in temperature of 10 °C, over a 4-week period (from 28.5 °C to 18.5 °C) to reduce cold shock which was followed by two rounds of radiation exposure over 10 days for a total whole-body dose of 0.32 Gy. RNA sequencing revealed differences in gene expression regulation in the gastrointestinal tract (GIT) between the impact of radiation exposure along with the induction of a “torpor-like state”, and their combination. The induction of hypothermia was phenotypically validated through reductions in metabolic rates and activity levels. Exposure to radiation was observed to cause DNA damage and oxidative stress, eliciting cellular responses such as activation of steroidal signaling pathways, alterations in metabolism, and arrest of the cell cycle. The induced hypothermic state mitigated these effects by promoting pro-survival signaling, reducing oxidative stress, and enhancing the detection and removal of misfolded proteins [36,37]. Radiation exposure in the liver disrupted lipid metabolism, absorption, wound healing, immune response, and fibrogenic pathways. Induced hypothermia, however, reduced metabolic activity while enhancing pro-survival, anti-apoptotic, and DNA repair pathways. When combined, the induced hypothermia mitigated radiation-induced damage by activating stress responses while preserving DNA repair, pro-survival, and anti-apoptotic processes [38]. Furthermore, transcriptomic analysis of zebrafish muscle tissue revealed that radiation exposure upregulated inflammatory and immune-related pathways, along with a regeneration phenotype mediated by *STAT3* and *MYOD1*, while downregulating DNA repair two days post-irradiation. Hypothermia increased expression of mitochondrial translation, especially oxidative phosphorylation genes, and downregulated expression of extracellular matrix (ECM) and developmental genes. In the hypothermic + radiation group, hypothermia mitigated radiation-induced inflammation and ECM gene expression while increasing expression of endoplasmic reticulum stress-related genes. Interestingly, a comparative genomics analysis was conducted with hibernating brown bears which identified shared cold tolerance mechanisms, including upregulation of protein translation, amino acid metabolism, and hypoxia responses, and downregulation of glycolysis, ECM, and developmental genes. These findings highlight conserved molecular responses to cold stress and radiation exposure across species [39]. These studies exploited the zebrafish’s capacity to lower their metabolism and body temperature in response to environmental changes. However, a crucial challenge in applying similar torpor induction techniques to homeotherms like humans lies in overcoming thermogenesis, the process that maintains constant body temperature regardless of external conditions. In the context of hibernating animals’ resistance to muscle and bone atrophy, further research is required to develop therapeutics that can mimic this phenotype.

Additional animal studies have confirmed that hypothermic states provide protective effects against radiation exposure. For instance, Musacchia et al., demonstrated that hypothermic states in hamsters [40] and hibernating squirrels [41] increased mean survival times after irradiation in comparison to normothermic/active controls. In addition, Puspitasari et al., revealed that these radioprotective effects are, in part, a result of an increase in the expression of protective DNA damage response genes involved in recognizing and repairing double-strand breaks [42]. In fact, induction of a hypothermic state has been shown to offer protective effects even when induced post-irradiation, suggesting active reparation mechanisms as opposed to passive protective mechanisms [43]. During torpor, a hypoxic cellular environment involving decreased cellular oxygen concentrations is achieved as a result of decreased respiration and circulation, and sustained by a reduction in metabolism to prevent oxygen starvation [44]. This may confer radio-resistance by decreasing the amount of reactive oxygen species (ROS) that can be generated after radiation exposure. This is known as the “oxygen effect,” which has been known to confer radio-resistance to solid tumors during radiotherapy [45]. In a study examining the effects of ionizing radiation on rats, animals were exposed to 3 Gy of X-rays under either normothermic conditions or synthetic torpor. The results, assessed 4 h post-exposure, indicated that synthetic torpor could reduce radiation toxicity. Histological analysis of the liver and testicles revealed less damage in rats irradiated during torpor compared to those at normal temperature. Furthermore, there was a notable downregulation of the ataxia telangiectasia mutated (*ATM*) gene in the liver of torpid rats. In the testicles, a significant downregulation of genes involved in DNA damage signaling was also observed during synthetic torpor, suggesting a protective mechanism activated by torpor against radiation-induced cellular damage [46]. Previous work, including that by Hrvatin and colleagues revealed that neuronal circuits in the medial and lateral preoptic area (POA) of the hypothalamus play a critical role in the regulation of torpor [47]. They found that activating these neurons could induce a torpor like state. However, activating such neurons without an invasive and direct manipulation would be required in a clinical setting. More recently however, advances have led to the discovery of a noninvasive, torpor-like state in rodents has been achieved using transcranial ultrasound stimulation targeting the same hypothalamic preoptic area (POA). Through closed-loop feedback, mice have been maintained in a hypothermic and hypometabolic state for over 24 h. This involved activating POA neurons, inhibiting thermogenic brown adipose tissue, and suppressing the dorsomedial hypothalamus. Single-nucleus RNA sequencing identified the transient receptor potential cation channel, subfamily M, member 2 (*TRPM2*) as a key ultrasound-sensitive ion channel for this effect. The method also induced hypothermia and hypometabolism in non-torpid animals like rats, highlighting its potential for safely inducing torpor-like states [48]. However, further animal work is needed to determine if this approach is scalable to humans. Interestingly, recent work by Uchino and colleagues found that inhalation anesthetics activate the same neurons in the POA region, leading to a reduction in body temperature [49].

### 3.2. The Targeted Temperature Management Model

While it remains the view of the scientific community that inducing a torpor-like state in humans should be based on replicating the physiological changes seen in hibernating animals through conserved mechanisms, the use of sedative agents along with active cooling has been proposed as an alternative approach. Targeted temperature management (TTM) is a medical treatment that involves sedation and cooling the body to between 32 and 34 °C, with patients being cooled at a rate of 1 to 2 °C per hour and a rewarming phase of 0.25 to 0.5 °C per hour to avoid adverse effects. It is the closest approximation in medicine to an induced torpor-like state, utilizing body temperatures like those observed in bears during torpor. TTM protocols have been safely used for up to 14 days in humans and are globally recognized as a standard of care to improve neurological outcomes, reduce tissue damage, and decrease mortality in patients suffering from cardiac arrest or neonatal hypoxic–ischemic encephalopathy [34,50,51]. Randomized clinical trials have demonstrated that TTM improves neurological outcomes, as measured by the cerebral performance category, and increases survival rates in cardiac arrest patients [52]. In ischemic events, anesthetics have been shown to reduce cerebral blood flow and oxidative metabolism in the brain in a dose-dependent manner, which is believed to contribute to their neuroprotective effects [53]. Additionally, hypothermia is understood to mitigate excessive glutamate release in the brain, reduce ion pump dysfunction that can lead to elevated calcium release, and decrease excess leakage through capillaries [54]. In replicating a torpor-like state, managed hypothermia in humans reduces oxygen consumption by 6% in body tissues and 8–10% in the brain for each °C below normal, such that at 32°C bodily tissues and cerebral metabolic activities can be reduced by up to 30% and 50% of their baseline metabolic rates, respectively [52]. A reduction in cellular oxygen concentration should therefore limit ROS generation upon exposure to radiation during space travel, protecting cells and DNA from oxidative damage. It has been shown that anesthesia alone can confer radioprotective effects by reducing metabolic activity and oxygen consumption, as demonstrated in studies by Mack, H.P., and Figge, F.H., 1952 [55]. These findings suggest that anesthesia leads to respiratory depression and reduced tissue oxygenation that may limit the production of ROS, mitigating radiation damage. While these effects are promising, combining anesthesia with active cooling to induce a deeper hypometabolic and hypothermic state could provide even greater radioprotective benefits. Hence, lowering body temperature will therefore decrease metabolic demands, suppress oxidative stress, and enhance DNA repair mechanisms, as shown in models of hypothermia and torpor. The synergy between reduced metabolism and controlled hypothermia therefore represents a compelling strategy for replicating the radioprotective and neuroprotective states observed in hibernating animals, offering potential applications for long-term spaceflight and other extreme conditions. Furthermore, we know that hypothermia is also known to reduce respiration, and the heart rate can be reduced to from 60–100 beats/min to 40–45 beats/min at 32 °C. This heart rate is considered normal at this temperature and does not require treatment as the decrease in metabolic rate is equal to or greater than the decrease in cardiac output. Although, a further drop in body temperature below 30 °C increases the risk of tachyarrhythmias such as atrial fibrillation and should therefore be avoided [54,56]. If an induced torpor-like state can be proven beneficial to the health of astronauts against the challenges of spaceflight then clinically used anesthetics, which have been well studied and characterized, may play a central role in its induction and maintenance for prospective long-term voyages. This view is consistent with the model presented in the SpaceWorks report [29] and a view supported by Regan et al., who propose the use of anesthetics to achieve shallow metabolic depression for during space travel [57].

## 4. Anesthetics

**Figure 2:**
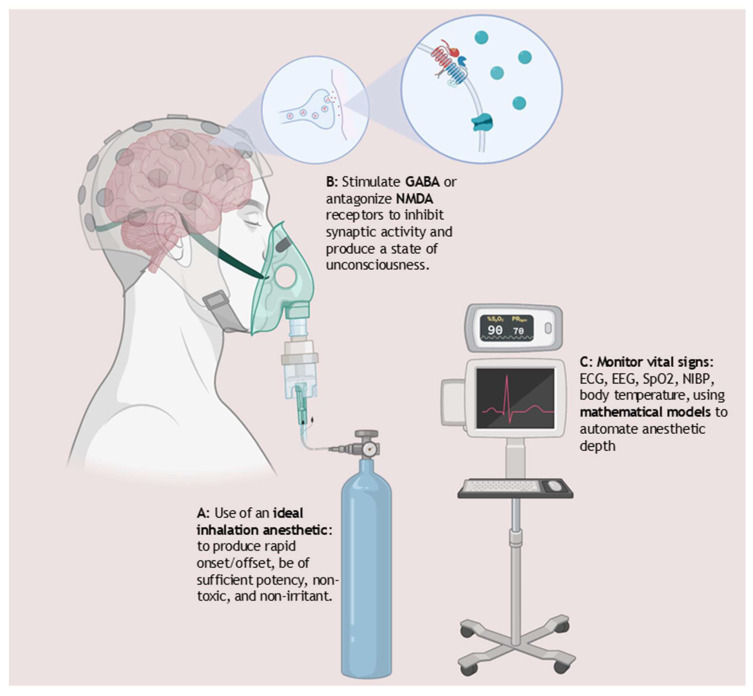
Schematic illustrating the use of an ideal inhalation anesthetic to suppress brain activity by interacting with receptors to induce unconsciousness, while monitoring crucial vital signs to automate the regulation of anesthetic depth. Created with BioRender.com (accessed on 26 April 2025).

Modern anesthetics are used in clinical practice and emergency departments to induce and maintain a state of controlled and reversible unconsciousness enabling the conduction of invasive medical procedures. Most general anesthetics act on the central nervous system to induce unconsciousness by stimulating the GABA receptor complex allowing Cl^−^ ions to flow into the nerve, leading to hyperpolarization, however some have been reported to antagonize NMDA receptors (Figure 2). One study found that isoflurane, for instance, inhibits the transmission of high-frequency electrical signals in the brain. It reduces the release of synaptic vesicles at presynaptic neurons by decreasing calcium influx through calcium ion channels, resulting in measurable and quantifiable changes in brain waves that correlate with the depth of anesthesia [58]. A systematic review of anesthetics used for sedation during TTM, which included 44 studies and protocols from 68 ICUs, found that midazolam and propofol were the most administered sedative agents. However, the review also highlighted that existing guidelines did not specify the optimal drug combinations for TTM, resulting in variability in the doses used [59].

The SpaceWorks report which presents TTM as a model of induced torpor proposed the use of Dexmedetomidine as a sedative agent due to the low risk of respiratory depression and neurocognitive dysfunction [29]. However, this work does not detail other potential anesthetic agents, or the properties or qualities that might need to be considered when repurposing these drugs. We therefore aim to introduce the reader to some fundamental properties of inhalation anesthetics to promote discussion on their suitability for advancing and achieving a torpor-like state based on a targeted temperature management model.

### 4.1. Properties of Inhalation Anesthetics

The most commonly used technique for maintaining general anesthesia in the USA is through the use of inhalation anesthetics (IA) [60]. Many of these inhalational anesthetics (IAs) are volatile agents that need to be vaporized before inhalation. The most common volatile agents include the fluorinated ethers; isoflurane, sevoflurane, and desflurane. Nitrous oxide (N_2_O) and Xenon, on the other hand, are already in a gaseous state and do not require vaporization. There are several qualities that make an inhalation anesthetic an attractive sedative. For practical application, IAs should possess a pleasant odor and should be easy to administer using vaporizers specifically calibrated for each type of inhalation agent. Additionally, these anesthetics need to be chemically stable and non-flammable. It’s important to note that vaporizers are designed to be agent-specific and are not interchangeable between different anesthetics. Vaporizers operate on the principle of converting liquid anesthetics into gas and then controlling the delivery by adjusting the ratio of vaporization to fresh gas flow. Clinically, they should possess adequate potency, allow for rapid induction and recovery from anesthesia, be non-irritating and non-toxic (as shown in Figure 2), and exert minimal depression on the cardiac and respiratory systems. If inhalation anesthetics are employed in a model of induced torpor for extended periods, some of these qualities may become even more critical [61].

#### 4.1.1. Potency

**Figure 3:**
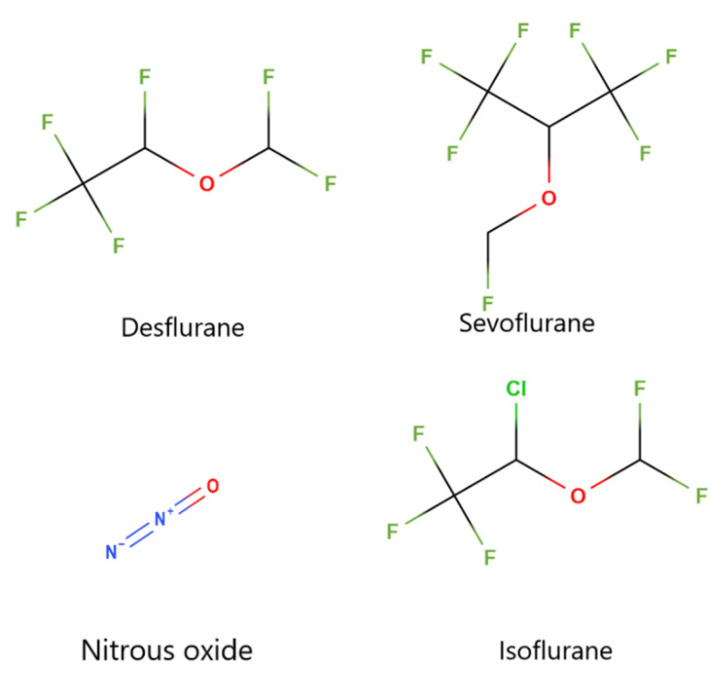
Chemical structures of commonly used inhaled anesthetics: Desflurane, Sevoflurane, Isoflurane, and the gas Nitrous Oxide. Fluorine atoms are shown in green, oxygen atoms in red, nitrogen atoms in blue, and chlorine in green (for Isoflurane). Carbon atoms are implied at the intersections of bonds. Generated using Molview [70,71].

The physical properties of IAs can influence their potency. Many IA’s are small, halogenated compounds attached to fluorine, bromine, or chlorine atoms (see Figure 3 for molecular structures). Higher-molecular-weight compounds carrying heavier halogen atoms such as chlorine or bromine are correlated with increased potency, for example isoflurane. In contrast, lower molecular weight compounds such as desflurane and sevoflurane carry lighter halogen atoms such as fluorine, are less potent [62,63,64].

Additionally, an IA’s potency can be measured by its lipid solubility whereby more lipophilic gases that have a lower oil/gas co-efficient, possess greater potency. The most used measure of potency, however, is the minimum alveolar concentration (MAC), which describes the amount of vapor needed to render 50% of spontaneously breathing patients unresponsive to pain stimulation. More potent gases are expressed with a lower MAC, which is inversely related to the oil/gas co-efficient [64].

When selecting a suitable sedative for long-term use, it might seem that a more potent IA would be beneficial, as it would require smaller doses to achieve sedation, thereby reducing toxicity. However, the selection process is more complex, as some highly potent IAs with higher molecular weights have been associated with increased toxicity, as discussed below [65,66].

In addition, Chamorro et al., suggest that the pharmacokinetics of intravenous (IV) anesthetics such as propofol and midazolam may be altered under hypothermic conditions, leading to increased plasma concentrations that may prolong or deepen sedation and lead to increased risk of side effects [59]. Similarly, animal research [67] and results from studies on the use of IA in children [68] show that hypothermia reduced the MAC of these anesthetics thereby increasing their potency, as determined by the Dixon up-and-down method. This means that smaller doses may be required to achieve sedation [68,69].

#### 4.1.2. Onset/Offset

The onset/offset of an anesthetic relates to how rapidly anesthesia is achieved after administration and how quickly recovery takes place when removed. The onset/offset directly relates to the gas’ solubility in blood. Once absorbed in the lungs, the gas distributes according to pressure gradients, where low-blood-soluble gases (with low blood/gas co-efficient) achieve tensions and equilibrate more quickly, providing the driving force for entering the brain [72]. In other words, highly blood-soluble drugs are less able to diffuse out of the blood and have a slower onset of action, whereas less-soluble agents are quicker to diffuse toward the site of action. Molecular properties also impact the onset and offset of anesthetics. Highly fluorinated molecules, such as sevoflurane and desflurane, exhibit lower solubility, which leads to a faster onset and offset of anesthesia [73]. The onset of anesthesia can be accelerated by using a higher concentration of inspired gas, facilitated by increased fresh gas flow, which raises alveolar concentrations [74]. The onset and offset of an inhalation anesthetic are crucial factors for long-duration missions, as a faster onset generally leads to quicker recovery from the drug. This is particularly important when considering the need for crew arousal in response to emergency situations. Additionally, a faster offset is associated with shorter recovery times, which could reduce the need for early arousal and allow for extended use of an induced torpor-like state during interplanetary transit. This would help minimize the need for resource-intensive weight and maximize the protective benefits of the torpor state, as outlined by Bradford et. al. [29]. Conversely, in relation to hypothermia and anesthetic solubility, Zhou et al., observed that IA’s become more soluble in the brain, heart, muscle, liver, and fat tissue under hypothermic conditions, resulting in increased tissue/blood partition coefficients as a consequence of increased tissue capacity, indicating that a delay in recovery from anesthesia may occur [75]. Hence, the ideal anesthetic should be lowly soluble in blood and tissue, minimizing the risk of accumulating in body cavities, which can lead to complications such as compression. This characteristic is particularly critical in space travel, where changes in body fluid distribution due to microgravity can alter how anesthetics are handled by the body. In the unique environment of space, agents like nitrous oxide (N_2_O) could pose significant risks. For example, in patients with COPD on Earth, N_2_O’s low solubility in blood allows it to expand into body cavities, potentially leading to lung barotrauma. In space, similar dynamics could affect astronauts, making the choice of anesthetic agent especially crucial to avoid exacerbating health issues in an already challenging environment.

### 4.2. Anesthetic Uptake

Anesthetic uptake and distribution are crucial in determining the appropriate dose and can be influenced by factors such as body fat and age. The dosing of propofol, for example, a common anesthetic agent, varies depending on several factors, including the patient’s age, weight, and the specific medical conditions involved. For healthy adults, the recommended induction dose of propofol is typically between 1.5 to 2.5 mg/kg, administered intravenously over 20–30 s. This dosage may be adjusted downward for elderly patients who are more sensitive to the effects of anesthetics. For maintenance of anesthesia, propofol is usually administered through a continuous infusion starting at 100 to 200 mcg/kg/min or through intermittent boluses of 20–50 mg as needed [76]. Mathematical models have also been developed to predict anesthetic uptake by different tissues, which relies mainly on anesthetic solubility, tissue volume, and rate of blood flow to that tissue. Similarly, methods like the Dixon up-and-down approach, commonly used in anesthetic research to determine minimum alveolar concentrations, could be valuable for estimating anesthetic doses in targeted temperature management (TTM) [77]. Blood flow and tissue solubility dictate the distribution and uptake of inhaled anesthetics to various tissues which each inhaled anesthetic presents with different partial pressures where steady state blood gas tensions are achieved. IAs are highly soluble in the vessel-rich group of tissues, including the brain, kidneys, heart, liver, and endocrine organs. These tissues, characterized by high blood flow and relatively small volume, achieve steady-state blood gas tension more rapidly. Muscle, with a large volume but low perfusion rate, is next to achieve a steady state blood gas tension, while IAs are most soluble in fatty tissue but have poorer perfusion, taking days to approach a steady state [78]. In the future, with human models of an induced torpor-like state, it may be possible to exploit an individual’s own fat reserves as an energy source, as seen in hibernating animals, to supplement energy and nutrients delivered intravenously. Thus, it may be important to factor in changes in body composition over time when determining the appropriate anesthetic dose. Moreover, spaceflight leads to physiological changes such as an increase in cardiac output, reduced cerebral blood flow, changes to arterial pressure, and cardiovascular deconditioning [79,80] which may impact the efficacy of the anesthetics. Additionally, microgravity-induced fluid shift causes blood to pool in the head and torso, which might work to increase anesthetic concentration at the brain. Measuring physiological responses in patients anesthetized during Trendelenburg positioning, common during lower abdominal surgery, might provide insights into how fluid shift affects anesthesia and provide estimates for the required dose in a microgravity environment.

## 5. Metabolism

The rate of metabolism of an inhaled anesthetic also directly influences its toxicity and will likely be a limiting factor for long-term use during space travel. All the halogenated inhalation anesthetics are metabolized to varying degrees in the liver by cytochrome P-450, resulting in the production of toxic metabolites that can cause liver injury. In contrast, desflurane and sevoflurane have been associated with fewer cases of liver damage [65]. Table 1 outlines the most used IAs with their respective measures of potency, onset/offset, and toxicity. Notably, those halogenated compounds that undergo less metabolism in the liver are less potent but have a faster onset. Thus, selection of a halogenated IA comes with a trade-off between molecules having faster onset/offset (smallest blood/gas coefficient), being less potent (small oil/gas co-efficient and high MAC), but possessing lower toxicity (least metabolized) [64]. Finally, induced hypothermia slows the rate of metabolism of drugs metabolized by the cytochrome P450 system, which may affect IA clearance and result in increased toxicity. Selection of an IA should therefore reflect this, where less metabolized IAs would be deemed more suitable [81].

### 5.1. Considerations for Inhalation Anesthetics

Sevoflurane is a sedative agent halogenated by fluoride ions and requires administration through a calibrated vaporizer. It was discovered in the 1970s and approved for usage in the 1990s. Sevoflurane is known for its safety and versatility in clinical practice due to its non-pungent nature which makes it ideal for the induction of anesthesia. While possessing the properties of an airway irritant these effects are observed less than with Desflurane [83]. Several studies have been conducted to understand the mechanism of action with proposals that this inhalation agent induces inhibitory postsynaptic channel activity, gamma-aminobutyric acid (GABA) and glycine, and inhibits excitatory synaptic channel activity (N-methyl-D-aspartate (NMDA), nicotinic acetylcholine, serotonin, and glutamate in the central nervous system [61,83]. It has a lower blood/gas co-efficient than isoflurane resulting in a faster onset and quicker recovery from anesthesia, and while the blood/gas co-efficient is lower in desflurane, the difference was not found to translate into a faster discharge time in patients [84]. While administering sevoflurane can cause side effects related to respiratory depression and a dose-dependent decrease in arterial blood pressure, it does not affect cardiac output [74]. Thus, it has been demonstrated to have less of an impact on hemodynamic and cardiovascular parameters than desflurane and isoflurane, resulting in comparatively lower morbidity and mortality [85]. Additionally, co-administration with nitrous oxide produces less of a decrease in blood pressure and, unlike desflurane, rapid increases in sevoflurane concentration do not lead to increased heart rate [86]. Additionally, sevoflurane metabolism produces Compound A, which has been linked to renal toxicity in rats. However, a meta-analysis found that its clinical use did not result in significant increases in renal function biomarkers, such as creatinine and blood urea nitrogen levels, in healthy patients. However, clinical recommendations suggest avoiding sevoflurane in patients with existing renal dysfunction [87]. One potential drawback of sevoflurane compared with the more potent, but hemodynamically stable, desflurane is the reported risk of neurotoxic effects. Sevoflurane use has been associated with long-term cognitive impairment, mediated by neuroinflammation, neurotransmitter imbalance, and a reduction in brain-derived neurotrophic brain factor, reviewed further by Wang, et al. [88].

Desflurane presents as an attractive IA due to low blood solubility, resistance to chemical degradation, low toxicity, and negligible metabolism [89]. It was initially discovered in the 1970s and requires an inhalational vaporizer for administration. Like other inhalational agents, the mechanism of action is still not entirely known for desflurane. However, studies have shown a role in the positive and negative allosteric modulation of GABA_A_ [90]. It has the lowest blood solubility of the volatile IAs, with a blood/gas partition coefficient of 0.42, which imparts a faster onset/offset [64,91]. Its offset is also approximately 2–2.5 times faster than that of isoflurane, for example. This fast onset/offset leads to the ability to precisely control anesthetic depth [89] and would equally lead to a faster recovery with reduced rehabilitation. Additionally, it has the lowest hepatic metabolism of halogenated IAs, resulting in low levels of toxic metabolites. However, desflurane is not typically used during the induction of anesthesia despite favorable characteristics due to airway irritant effects that can cause coughing, apnea, laryngospasm, copious secretions, and excitatory movements. It is therefore more commonly used to maintain anesthesia after induction with intravenous sedatives such as propofol [92]. It can, however, be administered without a greater incidence of airway irritation than non-irritant IAs such as sevoflurane by means of a laryngeal mask. Additionally, the risk of airway irritation is minimized, as airway irritation occurs in concentrations exceeding 1 MAC, and maintenance usually does not require concentrations that exceed 1 MAC [93]. Desflurane, along with other halogenated agents, can decrease systemic arterial resistance and mean arterial pressure in a dose-dependent manner. It has also been attributed to causing increases in heart rate, along with other IAs such as isoflurane. However, the increase in heart rate is associated with a MAC > 1 and is compensated for by a decrease in cardiac output [85]. Additionally, studies have found that heart rate did not change when a MAC of 0.83 was used for maintaining anesthesia with desflurane [94].

Xenon is an inert noble gas reported for use as an anesthetic agent in two patients in 1951, showing promising results. Its mechanism of action for inducing anesthesia arises from the inhibition of NMDA receptors [95]. Xenon gas exhibits a number of ideal properties for an anesthetic, although its expensive production has limited its use [74]. While it is not the most potent IA with a MAC of 71 when administered with oxygen, it can produce anesthesia, and it is, for example, 1.5 times more potent than nitrous oxide. It does, however, possess the lowest blood solubility of the anesthetics discussed, with a blood/gas co-efficient of 0.12, imparting a faster onset and recovery than the likes of sevoflurane or isoflurane in combination with nitrous oxide and propofol [96,97,98]. One of its main advantages, particularly when it comes to long-term use, is that it is excreted through the lungs and does not undergo hepatic metabolism, and thus, toxic metabolites are not produced. Other advantages of xenon anesthesia include stable hemodynamic and respiratory parameters. For example, studies have shown that xenon decreased indices of cardiac function less than nitrous oxide and maintained higher blood pressure than propofol [99]. In contrast to other IAs such as desflurane and isoflurane, it causes a decrease in heart rate and does not lead to changes in mean arterial pressure, systemic vascular resistance, or cardiac output [74]. Neuroprotective effects of xenon in combination with therapeutic hypothermia have also been demonstrated in animal models [100], and its use has been found to improve mean arterial blood pressure during the cooling and rewarming phases of therapeutic hypothermia [100]. It is therefore likely that xenon will appear as a frontrunner in the selection of IAs for an induced torpor-like state, aided by its superior onset and recovery from xenon-induced anesthesia, non-toxicity, and stable hemodynamic profile.

### 5.2. Multimodal Anesthesia

It may be necessary, as seen in clinical practice, to use IV anesthetics in conjunction with inhalational anesthetics, given that some IAs possess tendencies to irritate respiratory airways. The use of both IVs and IAs in combination allows for the advantages of both types of agents to be utilized while reducing the required anesthetic dose and minimizing their individual side effects; this combined approach is known as “balanced anesthesia”. On the other hand, IV anesthetics are often used only for anesthetic induction, followed by the administration of IAs for maintenance. Commonly used IV anesthetics include those that enhance GABA activity in the CNS (etomidate, midazolam, propofol, and thiopental), those that antagonize NMDA receptors (ketamine), and those that stimulate opioid receptors [101]. Much like IAs, the ideal IV anesthetic should have a rapid on-set/off-set, be non-toxic, and chemically stable, and have minimal cardiac and respiratory effects. Similarly, pharmacokinetics would need to be considered in relation to their distribution, metabolism, and toxicity. However, if the IV anesthetics are to be used solely for the induction in achieving an induced torpor-like state and not over long durations, then these considerations become less of an issue.

Ketamine, on the other hand, produces a dissociative anesthesia where patients appear to be awake but are unresponsive to stimuli. However, it can have undesirable psychotomimetic (dissociative) effects where patients can experience hallucinations upon the emergence from anesthesia. Moreover, while it has effects on the respiratory system except for bronchodilation, it has been shown to increase heart rate and blood pressure. In this context, where it is expected that the heart rate will decrease in line with a reduced body temperature during an induced torpor-like state, ketamine use may be counterproductive; however, it is commonly used in combination with propofol “ketofol” in mitigating the depressive hemodynamic effects during anesthesia delivery [102,103].

Dexmedetomidine is a highly selective alpha-2-adrenergic agonist, used primarily for sedation as an adjunct therapy for general anesthetics. It was chosen as the preferred sedative in the SpaceWorks report [29] due to its minimal respiratory depression effects along with its anxiolytic, analgesic, and potential neuroprotective effects. Moreover, its use shortens the duration of mechanical ventilation and reduces the risk of delirium, so its use may be preferred over midazolam or ketamine for example [104]. Its use may also prove advantageous in the context of induced torpor, as it reduces the shivering response to mild hypothermia [105,106]. However, while this agent can lead to bradycardia and hypotension due to its sympatholytic effects, studies examining the effects of TTM and the incidence of bradycardia have shown that bradycardia can be recovered by reducing the infusion rate [107].

Propofol is one of the most widely used IV anesthetics for inducing anesthesia as it is potent, and has a quick onset of action owing to its high lipophilicity, allowing rapid distribution to the central nervous system. It is also rapidly metabolized, which increases total body clearance [108]. However, propofol administration is known to cause pain at the injection site and can lead to a decrease in arterial blood pressure and systemic vascular resistance, resulting in hypotension [109]. Moreover, it has been shown to cause dose-dependent respiratory depression [110]. Prolonged use of propofol can also lead to propofol infusion syndrome when administered > 4 mg/kg/h for more than 24 h, characterized by metabolic acidosis, hyperkalemia, hyperlipidemia, and rhabdomyolysis [110]. Hence, its use may be best suited solely for induction as opposed to a balanced anesthesia approach. Furthermore, it was previously assumed in clinical circles that the hemodynamic effects of propofol could exacerbate hemodynamic instability during TTM. According to the European Resuscitation Council Guidelines, bradycardia may be left untreated during TTM as long as blood pressure, lactate levels, ScvO_2_, and SvO_2_ remain within acceptable ranges [111]. One study found that although blood pressure decreased 30 min after propofol administration during TTM, the absence of a need for vasopressors indicates that propofol can be safely used during TTM. Statistical analyses in this study indicated that temperature was the only covariate independently associated with mean arterial pressure, suggesting a temperature-dependent effect that is typical at lower body temperatures [112]. Furthermore, the authors reference a study indicating that bradycardia during TTM for cardiac arrest treatment is associated with lower mortality rates, suggesting it may have protective effects that promote a desirable outcome [113].

Conversely, the GABA agonist etomidate has a fast onset and short duration of action, and it is often used for anesthetic induction due to its minimal effects on both hemodynamics and the respiratory system. In individuals who are predisposed to hypotension, it may be necessary to minimize further exacerbation. One study found that a combination of propofol + ketamine was not superior to low-dose etomidate in preserving hemodynamics [114]. However, evidence for its use during TTM is limited, and further work is needed to assess its hemodynamic effects with lower body temperatures. Furthermore, the long term use of etomidate raises the effect of cortisol production in the adrenal cortex [115].

### 5.3. Potential Side Effects of Anesthesia

The side effects of anesthesia, including the suppression of natural reflexes such as maintaining moist mucosal surfaces, are important considerations in medical practice. Both cholinergic and anticholinergic medications are effective in managing the side effects associated with secretions. Glycopyrrolate is commonly used to address excessive secretions, ensuring a more controlled and sterile environment, which is crucial in sensitive settings such as during surgery. Adequate hydration of mucosal surfaces can be maintained using devices like breathing tubes or other forms of tubing that help preserve the integrity of mucosal tissues [116].

Advancements in biomedical materials are also noteworthy. Materials designed to be immunologically inert are being developed to enhance compatibility and reduce the risk of immune system stimulation when used internally, such as in grafting applications. These materials are designed to be well-tolerated within the body, potentially reducing complications associated with foreign body reactions.

In scenarios where respiratory mechanics are compromised, providing oxygenation might be necessary through techniques like extracorporeal membrane oxygenation (ECMO). However, it’s crucial to maintain respiratory movement to prevent conditions such as atelectasis, which can occur when the lung collapses due to inactivity. Continuous respiratory activity is essential, following the musculoskeletal principle of “use it or lose it”. Devices like the iron lung might inspire the development of compact, efficient respiratory support systems suitable for space missions. These systems would need to be adapted to function in reduced gravity, ensuring astronauts’ lungs are effectively ventilated to prevent atelectasis and other complications in an environment where normal movement is restricted.

These adaptations would require innovative engineering and medical research to create systems that are both effective and feasible for use in the challenging environment of space. Such technologies could also have dual uses on Earth, particularly in remote or resource-limited environments where similar constraints to space exist.

In the context of space travel, there’s significant concern regarding the potential exacerbation of radiation-induced cataract formation due to anesthesia. Studies suggest that astronauts are exposed to cosmic radiation, which can increase the risk of cataracts, a risk that might be influenced by the administration of anesthesia. Research has highlighted the mechanisms involved in radiation-induced cataracts and the influence of environmental factors such as cosmic radiation on cataract development [117,118,119]. The research indicates that ionizing radiation can induce complex biological and mechanistic changes in the ocular lens, promoting cataractogenesis. Moreover, a study examining the lenses of astronauts with upgraded imaging technology found increased opacity values, especially in regions of the lens most susceptible to radiation damage [120]. These findings underscore the importance of developing protective strategies and adjusting medical protocols to mitigate such risks during long-duration space missions.

Anesthesia can exacerbate the development of cataracts primarily through its influence on oxidative stress within the body. During anesthesia, there may be an increase in ROS, which are known to cause oxidative damage to cellular components, including those in the lens of the eye, potentially accelerating cataract formation. This oxidative stress can lead to the deterioration of lens proteins and lipids, contributing to the clouding characteristic of cataracts. Moreover, certain anesthetics impact blood flow and oxygenation, potentially exacerbating oxidative stress or leading to conditions conducive to cataract development, such as hypoxia. Additionally, anesthesia might indirectly influence inflammatory responses, further promoting an environment that could hasten cataractogenesis [121,122,123].

To mitigate these risks, understanding the molecular pathways involved in oxidative stress during cataract development is crucial. This includes focusing on key mechanisms like mitochondrial dysfunction, endoplasmic reticulum stress, and various cell death pathways, particularly in lens epithelial cells. Emerging therapeutic strategies targeting these pathways with antioxidants and other pharmacological agents are being explored to prevent and manage cataract formation effectively.

## 6. Monitoring

Long-term sedation during space travel will require the development of systems that can automatically regulate the maintenance of unconsciousness in real-time and in response to physiological traits to prevent overdose, early arousal, or other adverse effects. Recommendations published by the American Society of Anesthesiologists recommend the monitoring of ECG, SpO_2_, NIBP, end-tidal CO_2_, and temperature [124]. During general anesthesia, inspired and expired oxygen should be measured as well as waveform capnography to measure the partial pressure of carbon dioxide, particularly during tracheal intubation, as decreased CO_2_ can lead to respiratory alkalosis and hypocapnia [125]. The ASA recommends monitoring of end-tidal anesthetic concentration (ETAC), which is a measurement of the arterial blood partial pressure important for inhaled anesthetics and a good indicator of unconsciousness. In IA use, maintaining an ETAC > 0.7 age-adjusted MAC is necessary to reduce accidental awareness [124]. However, this requirement might differ under hypothermic conditions, as MAC decreases. Furthermore, the ASA suggests using processed electroencephalogram (pEEG) to measure brain electrical activity, as anesthetics can alter brain waves. The use of pEEG to generate a bispectral index (BIS) during anesthesia, while limited in the USA, has successfully reduced the incidence of accidental awareness [126]. Notably, the use of mid-latency auditory evoked potentials has shown more utility in measuring anesthetic depth during xenon anesthesia. Bradford et al. suggest that systems such as the LifeGuard^®^ and BioHarness^®^ that are already used to monitor the health of astronauts be equipped to measure the necessary vital signs that will be of importance during an induced torpor-like state [29]. It may also be necessary, due to the effects of hypothermia, to monitor for cold-induced diuresis, which can cause tubular dysfunction if left untreated. Additionally, checking for electrolyte abnormalities in magnesium, potassium, and phosphate will be necessary, as decreased levels of these electrolytes can cause cerebral and coronary vasoconstriction, tachyarrhythmias, or respiratory muscle weakness [125]. It will also be important to monitor carbon monoxide levels if volatile anesthetics are used, which can be produced when they react with desiccated CO_2_ absorbent. This effect is greatest with desflurane, larger concentrations of IA, and at increased temperature. CO production may be reduced during hypothermia, and it is recommended that soda-lime be hydrated with 4.8% or more water to reduce CO production [127]. Total Parenteral Nutrition (TPN) will likely be necessary to meet astronauts’ daily nutritional needs. TPN must be tailored to an individual’s specific requirements, necessitating the monitoring of parameters such as electrolyte profiles, liver enzymes, and blood sugar levels. This monitoring is essential for adjusting TPN accordingly to avoid complications. For example, one complication associated with TPN use is parenteral nutrition-associated liver disease, which arises due to lipid overload [128]. Delivery of TPN will be further complicated during the hypothermic conditions of an induced torpor-like state, as it can hinder insulin sensitivity and reduce insulin secretion, resulting in hyperglycaemia. Blood glucose levels should therefore also be monitored, and insulin therapy might be required in managing blood glucose levels to avoid the adverse effects of hyperglycemia [129]. Thus, monitoring these parameters will likely involve the use of an arterial blood line to accurately measure blood chemistry. Challenges also arise with TPN storage, as amino acids, for instance, have been found to degrade over time, impacting the nutritional status of astronauts [130].

Anesthetics undergo metabolic processes that produce byproducts, which can be toxic if accumulated in high concentrations. In the confined and unique environment of space, where the body’s metabolic and detoxification processes may behave differently, it will be essential to monitor these toxic metabolites to prevent potential health issues. Regular and comprehensive blood analyses will be necessary to detect and quantify these metabolites. This monitoring can help identify early signs of toxicity, allowing for timely intervention and adjustment of anesthetic protocols.

Inhalation anesthetics, commonly used in medical settings, have been associated with bone marrow depression, a condition where the bone marrow’s ability to produce blood cells is impaired. This issue is particularly concerning for space missions due to the potential for compounded health risks, including decreased production of red blood cells, white blood cells, and platelets. This reduction would significantly impact the immune system, making the astronaut more susceptible to infections and impairing their ability to heal from injuries. Furthermore, a decline in red blood cells would lead to anemia, causing fatigue and reduced physical performance, which are critical issues during demanding space missions. Furthermore, bone marrow depression can affect bone density, a concern compounded by the microgravity environment of space. Space travelers already face challenges related to bone density loss due to reduced mechanical loading in microgravity. The additional impact of anesthetics on bone marrow could exacerbate these issues, leading to an increased risk of fractures and other bone-related complications. Implementing robust monitoring systems that not only track standard physiological parameters but also measure levels of toxic metabolites is essential. Advanced diagnostic tools and regular blood tests will be needed to manage and adjust anesthetic use to minimize toxicity. Despite its apparent convenience for providing nutrition during induced torpor-like states, significant challenges must be overcome for its safe and effective use during space travel.

## 7. Reevaluating Neuromuscular Blockade in Light of Muscle Atrophy Concerns

In space, the absence of gravity leads to muscle atrophy and bone density loss, as the physical demands on the body are significantly reduced compared to Earth. This natural degradation of muscle and bone poses serious challenges for the health and functionality of astronauts both during and after their missions [131]. Neuromuscular blockade is a critical component in modern anesthesia and intensive care, enabling procedures and interventions that require temporary paralysis of skeletal muscles. It is employed during various medical procedures, including surgeries and mechanical ventilation, to ensure patient immobility and facilitate better surgical conditions [132]. The two primary classes of neuromuscular blockers are non-depolarizing agents, which inhibit neuromuscular transmission by blocking acetylcholine receptors at the neuromuscular junction, and depolarizing agents, which cause an initial muscle contraction followed by paralysis [133]. One significant concern with the use of neuromuscular blockers is their association with muscle atrophy. Prolonged or repeated use of these agents can contribute to a phenomenon known as disuse atrophy. Muscle disuse, resulting from the lack of voluntary movement, can lead to muscle fiber degradation, loss of muscle mass, and diminished muscle function. The underlying mechanism involves a decrease in muscle protein synthesis and an increase in muscle protein breakdown. The balance between these processes is disrupted during prolonged immobilization or paralysis, leading to a net loss of muscle mass. This condition not only affects the immediate recovery post-procedure but can also have long-term impacts on a patient’s overall mobility and quality of life. Given the risks of muscle atrophy and the challenges faced by muscles in the space environment, it is essential to explore alternative approaches that can reduce these negative effects. One promising avenue is the development and use of agents that promote muscle contractions without causing injury or paralysis. These agents could potentially help maintain muscle activity and prevent atrophy during procedures requiring muscle relaxation. Research into muscle-stimulating agents has shown potential in addressing muscle atrophy. These agents, unlike traditional neuromuscular blockers, are designed to enhance muscle contraction through different mechanisms. For instance, pharmacological agents that activate muscle growth pathways, such as the mTOR (mammalian target of rapamycin) pathway, could help counteract muscle degradation [134]. Similarly, compounds that mimic the effects of exercise-induced muscle growth might provide benefits in maintaining muscle mass and function. Another alternative is electrical stimulation of muscles. This technique involves applying electrical impulses to muscles to induce contractions. Electrical stimulation has been used in rehabilitation settings to prevent muscle atrophy in patients who are unable to perform voluntary movements. It offers a way to maintain muscle activity and mass during periods of immobilization or disuse [132].

Immobilization associated with neuromuscular blockade raises significant concerns about skin integrity. The lack of movement can lead to skin breakdown and the development of pressure ulcers, which are particularly challenging in a microgravity environment. To mitigate these risks, it will be crucial to design specialized beds or pods that accommodate the unique conditions of space. These designs must not only support the astronaut’s body in a way that prevents pressure points but also incorporate materials and technologies that enhance skin breathability and distribute pressure evenly. This proactive approach in bed or pod design is essential to safeguard the health and well-being of astronauts during prolonged periods of immobility induced by synthetic torpor.

## 8. Hypothermia and Torpor

As discussed above, the benefits of induced an induced torpor-like state arise because of the hypo-metabolic state that in humans, can be facilitated through hypothermic body temperatures. The controlled reduction and maintenance of hypothermia will therefore be paramount in exploiting these benefits and TTM represents a testing bed for optimizing the induced torpor-like state protocol. During TTM, anesthetic agents lower the body temperature by impairing thermoregulatory responses regulated in the hypothalamus by inhibiting vasoconstriction and causing vasodilation. The reduction in metabolic rate also facilitates hypothermia by reducing non-shivering thermogenesis. Anesthesia also leads to heat loss by reducing the shivering threshold by 2–3 °C, leading to a delayed thermoregulatory response [56,135]. Body heat is also naturally lost through radiation, convection, evaporation, and conduction, but plateaus at approximately 35.5 °C. However, the lack of convection in microgravity will likely affect heat loss. Therefore, using “hibernation” pods combined with scavenging systems may be necessary to generate airflow around the crew, facilitating convection to assist with heat loss and remove spent anesthetic [136]. There are three main methods of cooling, consisting of convection systems such as ice packs or infusion of cold saline solution, surface cooling systems that circulate cold air/fluid through blankets or pads that are wrapped around the patient, or intravascular cooling systems. Intravascular cooling systems use a closed loop or balloon catheter to circulate cooled saline through the femoral or subclavian veins, thereby cooling the blood via convection. These systems have the ability to record and control blood temperature in real-time with a computerized auto-feedback mechanism [125,137] and have been found to be most effective and reliable in reducing core temperature [125]. In some instances, the use of IAs can lead to malignant hyperthermia, which can be fatal if left untreated. The presentation of malignant hyperthermia has been associated with variants in the *RYR1* gene, and DNA testing can be conducted to determine susceptibility [138].

Shivering is a common side effect of TTM observed in 40% of patients [139] which may counteract the benefits of TTM by frustrating the reduction in body temperature through heat generation, increasing the metabolism and oxygen demand, and additionally causing cerebral metabolic stress by decreasing brain tissue oxygen tension [140,141]. Several medications can be administered to control shivering, such as anti-pyretic drugs, NSAIDs, opioids, α-agonists, 5-HT agonists, NMDA antagonists, and neuromuscular blockade (NMB) agents. Given that the threshold for the activation of the shivering response starts at 35 °C and ends at 33.5 °C, it might be possible to pharmacologically inhibit shivering during the cooling phase and cease treatment beyond the 33.5 °C threshold. Jain et al. recommend that shivering interventions begin at the initiation of targeted temperature management (TTM). Patients should receive an antipyretic agent, such as acetaminophen or an NSAID, every 4–6 h, standing doses of buspirone (30 mg) every 8 h, and magnesium sulfate intravenously (either bolus or continuous) to maintain a serum level of 3 to 4 mg/dL.

They also suggest the use of a shivering scale to guide further treatment based on shivering severity, involving the administration of dexmedetomidate or opioids (fentanyl or meperidine) for mild to moderate shivering or neuromuscular blockade agents such as rocuronium, vecuronium, pancuronium, or cistracurium for more severe shivering [141]. However, developing a unique anti-shivering strategy will need to be tailored to the chosen sedative and target temperature to prevent compounding adverse effects. Additionally, the timing of neuromuscular blockade agents must be carefully considered in an induced torpor-like state, especially if neuromuscular electrical stimulation is used to combat muscle atrophy or if the goal is to avoid the need for mechanical ventilation [142].

### Understanding the Phases of Torpor Induction

The induction phase marks the transition from a normal metabolic state to torpor, characterized by a substantial reduction in physiological activity [17,18,20]. This phase is crucial for preparing the body for the low-energy state associated with torpor. The primary goal during induction is to safely and effectively lower metabolic rates while minimizing potential stress on the astronaut. To induce a torpor-like-state, the body temperature of the astronaut must be gradually lowered. Controlled cooling methods, such as advanced thermal regulation systems, are used to achieve this. Rapid temperature changes can cause physiological stress, so a gradual approach will mitigate potential risks. Administering sedatives or anesthetics will facilitate the transition into torpor. During the induction phase, continuous monitoring of vital signs, including heart rate, blood pressure, and body temperature, will be essential. This will ensure that the transition into torpor proceeds smoothly and that any complications are promptly addressed. Once the astronaut has entered a torpor-like-state, the maintenance phase will involve sustaining this low-metabolism state for the desired duration. This phase is crucial for conserving energy and resources while ensuring that the astronaut remains stable and healthy throughout the mission. Regular monitoring of vital signs and physiological parameters will continue during the maintenance phase. This monitoring will ensure that the astronaut remains in a stable torpid state. The emergence phase is the transition from a torpor-like-state back to full metabolic activity. This phase is critical, as it involves gradually restoring normal physiological functions while avoiding the complications associated with reactivation [18]. Rewarming the body must be performed gradually to prevent shock and other adverse effects. Controlled rewarming methods, such as gradual increases in ambient temperature or the use of warming devices, are essential for a safe transition. Post-torpor rehabilitation protocols will be necessary to help the astronaut readjust to full metabolic function. This may include physical therapy, nutritional support, and monitoring for any residual effects from the torpor-like- state. Vital signs, cognitive function, and overall health must be carefully assessed to address any issues promptly.

## 9. The Implications of Prolonged Anesthetic Exposure in Space

As humanity ventures further into space, understanding the implications of prolonged exposure to anesthetics becomes increasingly critical. In the context of long-term space missions, the extended duration of exposure to anesthetics poses significant concerns due to the lack of comprehensive studies on their long-term effects. During extended missions, astronauts may be exposed to anesthetics for durations significantly longer than those typically encountered on Earth. While anesthetics are crucial for managing pain and ensuring comfort during medical procedures, their prolonged use in the unique environment of space introduces several concerns. The primary issue is the potential for long-term health effects that are not yet fully understood. On Earth, anesthetics are usually administered for short-term procedures, with post-operative recovery closely monitored [132]. In contrast, space missions could expose astronauts to anesthetics over months or even years, raising questions about cumulative effects on health and performance.

Current research on anesthetic exposure has predominantly focused on short-term administration. Most studies, including those involving intensive care unit (ICU) patients, have examined exposures lasting up to 30 days [132]. While this provides some insight, it falls short of addressing the extended exposure scenarios faced in space. The longest durations studied in ICU settings are relatively brief compared to the potential lengths of space missions. Consequently, there is a substantial gap in our understanding of how prolonged exposure to anesthetics affects the human body over extended periods. The limited data available raises concerns about possible long-term side effects, including impacts on cognitive function, cardiovascular health, and overall physiological stability. Without robust research on extended exposure, astronauts may be at risk of unforeseen complications that could jeopardize their health and mission success. Given the uncertainties surrounding the long-term effects of anesthetics, there is an urgent need for further research, particularly using animal models. Studies should focus on anesthetics such as Dexmedetomidine, Ketamine, and Propofol, which are commonly used in medical settings. These studies should aim to simulate prolonged exposure scenarios to assess potential long-term effects on various physiological systems. Animal models are valuable for such research because they can provide preliminary insights into how extended anesthetic exposure might affect human health. Additionally, these studies can help identify potential biomarkers for adverse effects and guide the development of safer anesthetic protocols for space missions. Understanding these effects will be crucial for designing effective countermeasures and ensuring that astronauts remain healthy and capable throughout their missions.

The advent of space exploration has brought about unique medical and technological challenges, particularly in the fields of airway management and anesthesia. The microgravity environment of space introduces complex issues that affect spontaneous ventilation under general anesthesia, such as aspiration and atelectasis, along with the efficacy of both invasive and non-invasive ventilation strategies. Addressing these challenges requires innovative adaptations in medical equipment, procedures, and environmental controls aboard spacecraft. In space, the lack of gravity affects the distribution of air and fluids within the human body, complicating spontaneous ventilation during anesthesia. This can increase the risk of aspiration, where fluids are inhaled into the lungs, and atelectasis, where alveoli collapse, leading to reduced lung function. Preventative strategies in space might include more stringent fasting guidelines before anesthesia and the use of elevated upper body positioning, similar to strategies used in terrestrial settings but adapted for microgravity.

Managing airway and ventilation in space is significantly complicated by microgravity. Traditional invasive ventilation techniques, which involve intubation, and non-invasive methods, like masks and nasal cannulas, need redesigning to ensure effective delivery and secure fitting. Furthermore, the spacecraft’s closed environment necessitates meticulous management of exhaled gases, including CO_2_ and anesthetic agents.

The design of ventilation circuits or pods must efficiently remove CO_2_ while conserving valuable anesthetic gases. Techniques such as exhaled gas recirculation could be adapted to minimize the loss of anesthetic gases, employing technologies that absorb and recycle these gases back into the system. Managing gas scavenging in space also involves ensuring that harmful anesthetic gases do not accumulate in the cabin, maintaining air quality, and reducing potential health risks to the crew.

The calibration of volatile anesthetic agent vaporizers is another challenge, as these devices are sensitive to barometric pressure, which varies significantly from Earth to space. Special vaporizer designs, such as those used for desflurane, which require heating to achieve the boiling point, must be adapted to function under both altered pressure conditions and the need for energy efficiency aboard spacecraft.

Addressing the medical needs of astronauts under anesthesia also involves the integration of life support systems that control temperature and humidity, essential for maintaining hemostasis and ensuring comfort and safety during medical procedures. Nutritional support and waste management systems must also be adapted to work seamlessly with medical care protocols, ensuring that astronauts remain healthy and that medical interventions do not compromise spacecraft environments.

Beyond managing anesthesia, promoting physical health through exercise regimes to prevent muscle atrophy and flexibility loss is crucial. Radiation protection is another critical consideration, requiring innovative materials and shielding strategies to protect astronauts from cosmic rays and other space-specific hazards, which could adversely affect their health.

Slowing down metabolism through medical hibernation could potentially reduce oxidative stress and the aging process, mirroring the benefits seen in natural hibernators like bears and rodents. Protecting astronauts’ gametes is also essential for long-term space missions, especially for those of reproductive age, to prevent genetic damage from radiation exposure.

As humanity edges closer to long-term interplanetary travel, the challenges of providing medical care in space, particularly concerning airway management and anesthesia, become more pertinent. Addressing these issues requires a multidisciplinary approach involving aerospace engineers, medical researchers, and space agencies, working together to develop solutions that ensure the safety and well-being of astronauts while pushing the boundaries of what is medically possible in space.

## 10. Conclusions

Spaceflight is associated with hazards including cosmic radiation exposure and microgravity, which impact different echelons of biological organization that span the molecular, cellular, and organ levels. These insults lead to adverse health outcomes that impair astronaut well-being. The Artemis program with the objective of returning to the moon, is hastening technological development that will transport humans to Mars and advance interplanetary space travel. However, the challenges presented by long-term space travel need to be addressed and appropriate countermeasures developed to protect astronaut health.

Hibernation is a seasonal heterothermy observed in nature across several species, primarily during winter months. It is characterized by low body temperature, reduced breathing and heart rate, and a minimal metabolic rate. Hibernating animals exploit this state as a survival mechanism to conserve energy during periods of food scarcity or cold temperatures. The concept that hibernation confers protective effects, particularly against radiation exposure, has prompted studies aiming to induce a torpor-like state in humans, which date back to the 1960s. Controlled targeted temperature management has been successfully exploited to lower mortality rates and improve outcomes in acute trauma and cardiac arrest patients, and is the closest we have in medical practice to an induced torpor-like state in humans. It might be possible to safely and reliably implement synthetic torpor in humans based on a TTM approach involving the use of modern anesthetics, which would provide a step-change advancement in space travel. It would reduce both the burden of life support, including oxygen, nutritional, and water requirements of astronauts. Additionally, it has the potential to protect against the harsh environment of space and eliminate the psychological stresses of long periods in space. While the aim of this manuscript is to promote discussion on the advantages and drawbacks of potential sedative candidates, Xenon emerges as a front-runner when considering inhalation anesthetics for producing an induced torpor-like state. Although it is costly to produce and not the most powerful IA, its non-toxic and hemodynamically stable qualities make it particularly well-suited for minimizing potential complications during long-term space missions.

As space agencies prepare for extended missions such as those proposed by the Artemis program, understanding and mitigating the physiological challenges faced by astronauts due to cosmic radiation and microgravity becomes imperative. These conditions impact biological functions across various levels, leading to significant health risks. One promising countermeasure being explored is the induction of a synthetic torpor-like state, akin to natural hibernation observed in wildlife. This state conserves energy and reduces life support requirements by lowering body temperature and metabolic rates. Targeted Temperature Management (TTM) techniques, borrowed from medical practices, could potentially be adapted to safely induce such states in astronauts using modern anesthetics like xenon, which is favored for its non-toxic and stable properties despite its cost and lower potency. As missions aim for more distant targets such as Mars, the integration of comprehensive health management strategies becomes critical. This includes advanced life support systems, real-time health monitoring, and rapid medical response capabilities. The pathway to implementing these advanced health management strategies in space travel involves multidisciplinary collaboration among space agencies, medical researchers, and technology developers to ensure that astronauts can safely reach and explore new frontiers. Each step forward in this research not only has the potential to revolutionize space travel but could also offer new insights and technologies beneficial for medical science on Earth.

## Figures and Tables

**Table 1:** Table showing the oil/gas co-efficient, MAC, blood/gas co-efficient and percentage of hepatic metabolism of the inhalation anesthetics [64,82].

	Potency		Onset/Offset	Toxicity
Anesthetic	Oil/gas Co-Efficient	MAC	Blood/gas Co-efficient	Metabolism
Xenon	1.9	71	0.12	0
Nitrous Oxide	1.4	104	0.47	0
Desflurane	19	6.35	0.42	0.02
Sevoflurane	53	2	0.65	5
Isoflurane	91	1.15	1.43	2

## Supplementary Materials

The following supporting information can be downloaded at: https://www.mdpi.com/article/10.3390/life15050706/s1, Table S1: Systematic_Review_Categories. Table S2: PRISMA_2020_checklist.

## References

[1] Lester D.F., Robinson M. (2009). Visions of exploration. Space Policy.

[2] Moroz V., Huntress W., Shevalev I. (2002). Planetary missions of the 20th century. Cosm. Res..

[3] Blumberg B.S. (2011). Astrobiology, space and the future age of discovery. Philos. Trans. R. Soc. A Math. Phys. Eng. Sci..

[4] Smith M., Craig D., Herrmann N., Mahoney E., Krezel J., McIntyre N., Goodliff K. The artemis program: An overview of nasa’s activities to return humans to the moon. Proceedings of the 2020 IEEE Aerospace Conference.

[5] Yatagai F., Honma M., Dohmae N., Ishioka N. (2019). Biological effects of space environmental factors: A possible interaction between space radiation and microgravity. Life Sci. Space Res..

[6] Hellweg C.E., Baumstark-Khan C. (2007). Getting ready for the manned mission to Mars: The astronauts’ risk from space radiation. Naturwissenschaften.

[7] Smith S., Abrams S., Davis-Street J., Heer M., O’Brien K., Wastney M., Zwart S. (2014). Fifty years of human space travel: Implications for bone and calcium research. Annu. Rev. Nutr..

[8] Stein T., Wade C. (2005). Metabolic consequences of muscle disuse atrophy. J. Nutr..

[9] Vana N., Hajek M., Berger T., Fugger M., Hofmann P. (2006). Novel shielding materials for space and air travel. Radiat. Prot. Dosim..

[10] Riva D., Rossitto F., Battocchio L. (2009). Postural muscle atrophy prevention and recovery and bone remodelling through high frequency proprioception for astronauts. Acta Astronaut..

[11] Davis S.A., Davis B.L. (2012). Exercise equipment used in microgravity: Challenges and opportunities. Curr. Sports Med. Rep..

[12] Williams D.R. (2002). Bioastronautics: Optimizing human performance through research and medical innovations. Nutrition.

[13] Heldmaier G., Ruf T. (1992). Body temperature and metabolic rate during natural hypothermia in endotherms. J. Comp. Physiol. B.

[14] Barnes B.M. (1989). Freeze avoidance in a mammal: Body temperatures below 0 C in an arctic hibernator. Science.

[15] Sonntag M., Arendt T. (2019). Neuronal activity in the hibernating brain. Front. Neuroanat..

[16] Ruf T., Geiser F. (2015). Daily torpor and hibernation in birds and mammals. Biol. Rev..

[17] Geiser F. (2021). Ecological Physiology of Daily Torpor and Hibernation.

[18] Griko Y., Regan M.D. (2018). Synthetic torpor: A method for safely and practically transporting experimental animals aboard spaceflight missions to deep space. Life Sci. Space Res..

[19] Geiser F. (2004). Metabolic rate and body temperature reduction during hibernation and daily torpor. Annu. Rev. Physiol.

[20] Boyer B.B., Barnes B.M. (1999). Molecular and Metabolic Aspects of Mammalian Hibernation: Expression of the hibernation phenotype results from the coordinated regulation of multiple physiological and molecular events during preparation for and entry into torpor. BioScience.

[21] Tøien Ø., Blake J., Edgar D.M., Grahn D.A., Heller H.C., Barnes B.M. (2011). Hibernation in black bears: Independence of metabolic suppression from body temperature. Science.

[22] Tøien Ø., Blake J., Barnes B.M. (2015). Thermoregulation and energetics in hibernating black bears: Metabolic rate and the mystery of multi-day body temperature cycles. J. Comp. Physiol. B.

[23] Cannon B., Nedergaard J. (2004). Brown adipose tissue: Function and physiological significance. Physiol. Rev..

[24] Serkova N.J., Rose J.C., Epperson L.E., Carey H.V., Martin S.L. (2007). Quantitative analysis of liver metabolites in three stages of the circannual hibernation cycle in 13-lined ground squirrels by NMR. Physiol. Genom..

[25] Dausmann K.H., Glos J., Ganzhorn J.U., Heldmaier G. (2004). Hibernation in a tropical primate. Nature.

[26] Srivastava A., Kumar Sarsani V., Fiddes I., Sheehan S.M., Seger R.L., Barter M.E., Neptune-Bear S., Lindqvist C., Korstanje R. (2019). Genome assembly and gene expression in the American black bear provides new insights into the renal response to hibernation. DNA Res..

[27] Choukér A., Ngo-Anh T.J., Biesbroek R., Heldmaier G., Heppener M., Bereiter-Hahn J. (2021). European space agency’s hibernation (torpor) strategy for deep space missions: Linking biology to engineering. Neurosci. Biobehav. Rev..

[28] ESA (2022). Hibernate for a trip to Mars, the bear way. Human and Robotic Exploration.

[29] Bradford J., Merrel B., Schaffer M., Talk D. (2018). Advancing Torpor Inducing Transfer Habitats for Human Stasis to Mars. Phase; II.

[30] Cheng L., Lisowska H., Sollazzo A., Wegierek-Ciuk A., Stepień K., Kuszewski T., Lankoff A., Haghdoost S., Wojcik A. (2015). Modulation of radiation-induced cytogenetic damage in human peripheral blood lymphocytes by hypothermia. Mutat. Res. Genet. Toxicol. Environ. Mutagen..

[31] Ignat’ev D., Fialkovskaia L., Perepelkina N., Markevich L., Kraev I., Kolomiĭtseva I. (2006). The effect of hypothermia on the rat radioresistance. Radiatsionnaia Biol. Radioecol..

[32] Musacchia X. (1973). Hibernation, Stress, Intestinal Functions, and Catecholoamine Turnover Rate in Hamsters and Gerbils. Patent Application.

[33] Barr R., Musacchia X. (1972). Postirradiation hibernation and radiation response of ground squirrels: Telemetry surveillance. Radiat. Res..

[34] Cerri M. (2017). The Central Control of Energy Expenditure: Exploiting Torpor for Medical Applications. Annu. Rev. Physiol..

[35] Dang L., Lisowska H., Manesh S.S., Sollazzo A., Deperas-Kaminska M., Staaf E., Haghdoost S., Brehwens K., Wojcik A. (2012). Radioprotective effect of hypothermia on cells—a multiparametric approach to delineate the mechanisms. Int. J. Radiat. Biol..

[36] Cahill T. (2023). A Systems Biology Approach to Dissect the Hazardous Effects of Spaceflight and Investigate Induced Torpor as a Therapeutic Countermeasure. Ph.D. Thesis.

[37] Cahill T., da Silveira W.A., Renaud L., Williamson T., Wang H., Chung D., Overton I., Chan S.S.L., Hardiman G. (2021). Induced Torpor as a Countermeasure for Low Dose Radiation Exposure in a Zebrafish Model. Cells.

[38] Cahill T., da Silveira W.A., Renaud L., Wang H., Williamson T., Chung D., Chan S., Overton I., Hardiman G. (2023). Investigating the effects of chronic low-dose radiation exposure in the liver of a hypothermic zebrafish model. Sci. Rep..

[39] Cahill T., Chan S., Overton I.M., Hardiman G. (2023). Transcriptome Profiling Reveals Enhanced Mitochondrial Activity as a Cold Adaptive Strategy to Hypothermia in Zebrafish Muscle. Cells.

[40] Musacchia X., Volkert W., Barr R. (1971). Radioresistance in hamsters during hypothermic depressed metabolism induced with helium and low temperatures. Radiat. Res..

[41] Musacchia X., Barr R. (1968). Survival of whole-body-irradiated hibernating and active ground squirrels; Citellus tridecemlineatus. Radiat. Res..

[42] Puspitasari A., Cerri M., Takahashi A., Yoshida Y., Hanamura K., Tinganelli W. (2021). Hibernation as a tool for radiation protection in space exploration. Life.

[43] Ghosh S., Indracanti N., Joshi J., Ray J., Indraganti P.K. (2017). Pharmacologically induced reversible hypometabolic state mitigates radiation induced lethality in mice. Sci. Rep..

[44] Boutilier R.G. (2001). Mechanisms of cell survival in hypoxia and hypothermia. J. Exp. Biol..

[45] Harada H. (2011). How can we overcome tumor hypoxia in radiation therapy?. J. Radiat. Res..

[46] Tinganelli W., Hitrec T., Romani F., Simoniello P., Squarcio F., Stanzani A., Piscitiello E., Marchesano V., Luppi M., Sioli M. (2019). Hibernation and Radioprotection: Gene Expression in the Liver and Testicle of Rats Irradiated under Synthetic Torpor. Int. J. Mol. Sci..

[47] Hrvatin S., Sun S., Wilcox O.F., Yao H., Lavin-Peter A.J., Cicconet M., Assad E.G., Palmer M.E., Aronson S., Banks A.S. (2020). Neurons that regulate mouse torpor. Nature.

[48] Yang Y., Yuan J., Field R.L., Ye D., Hu Z., Xu K., Xu L., Gong Y., Yue Y., Kravitz A.V. (2023). Induction of a torpor-like hypothermic and hypometabolic state in rodents by ultrasound. Nat. Metab..

[49] Uchino E., Kusumoto-Yoshida I., Kashiwadani H., Kanmura Y., Matsunaga A., Kuwaki T. (2024). Identification of hypothermia-inducing neurons in the preoptic area and activation of them by isoflurane anesthesia and central injection of adenosine. J. Physiol. Sci..

[50] Song S.S., Lyden P.D. (2012). Overview of therapeutic hypothermia. Curr. Treat. Options Neurol..

[51] Schaffer M., Bradford J., Talk D. A feasible, near-term approach to human stasis for long-duration deep space missions. Proceedings of the 67th International Astronautical Congress (IAC).

[52] Nordeen C.A., Martin S.L. (2019). Engineering human stasis for long-duration spaceflight. Physiology.

[53] Slupe A.M., Kirsch J.R. (2018). Effects of anesthesia on cerebral blood flow, metabolism, and neuroprotection. J. Cereb. Blood Flow Metab..

[54] Saigal S., Sharma J.P., Dhurwe R., Kumar S., Gurjar M. (2015). Targeted temperature management: Current evidence and practices in critical care. Indian J. Crit. Care Med. Peer-Rev. Off. Publ. Indian Soc. Crit. Care Med..

[55] Mack H.P., Figge F.H. (1952). Sodium Pentobarbital Anesthesia and Mortality from X-Radiation in C57 Black Mice. Science.

[56] Bindu B., Bindra A., Rath G. (2017). Temperature management under general anesthesia: Compulsion or option. J. Anaesthesiol. Clin. Pharmacol..

[57] Regan M.D., Flynn-Evans E.E., Griko Y.V., Kilduff T.S., Rittenberger J.C., Ruskin K.J., Buck C.L. (2020). Shallow metabolic depression and human spaceflight: A feasible first step. J. Appl. Physiol..

[58] Palanca B., Avidan M., Mashour G. (2017). Human neural correlates of sevoflurane-induced unconsciousness. BJA Br. J. Anaesth..

[59] Chamorro C., Borrallo J.M., Romera M.A., Silva J.A., Balandín B. (2010). Anesthesia and analgesia protocol during therapeutic hypothermia after cardiac arrest: A systematic review. Anesth. Analg..

[60] Nimmo A., Absalom A., Bagshaw O., Biswas A., Cook T., Costello A., Grimes S., Mulvey D., Shinde S., Whitehouse T. (2019). Guidelines for the safe practice of total intravenous anaesthesia (TIVA) joint guidelines from the association of anaesthetists and the society for intravenous anaesthesia. Anaesthesia.

[61] Alan R., Aitkenhead G.S., David R. (2007). Textbook of Anaesthesia.

[62] Targ A.G., Yasuda N., Eger E.I., Huang G., Vernice G.G., Terrell R.C., Koblin D.D. (1989). Halogenation and anesthetic potency. Anesth Analg..

[63] Taylor A., McLeod G. (2020). Basic pharmacology of local anaesthetics. BJA Educ..

[64] Pinnock C., Lin T., Smith T. (2003). Fundementals of Anaesthesia.

[65] Safari S., Motavaf M., Siamdoust S.A.S., Alavian S.M. (2014). Hepatotoxicity of halogenated inhalational anesthetics. Iran. Red Crescent Med. J..

[66] Wang Y., Ming X.X., Zhang C.P. (2020). Fluorine-Containing Inhalation Anesthetics: Chemistry, Properties and Pharmacology. Curr. Med. Chem..

[67] Vitez T.S., White P.F., Eger E.I. (1974). Effects of hypothermia on halothane MAC and isoflurane MAC in the rat. Anesthesiology.

[68] Liu M., Hu X., Liu J. (2001). The effect of hypothermia on isoflurane MAC in children. J. Am. Soc. Anesthesiol..

[69] Antognini J.F. (1993). Hypothermia eliminates isoflurane requirements at 20 degrees C. Anesthesiology.

[70] Bergwerf H. (2015). MolView: An attempt to get the cloud into chemistry classrooms. DivCHED CCCE Comm. Comput. Chem. Educ..

[71] Kim S., Chen J., Cheng T., Gindulyte A., He J., He S., Li Q., Shoemaker B.A., Thiessen P.A., Yu B. (2021). PubChem in 2021: New data content and improved web interfaces. Nucleic Acids Res..

[72] Becker D.E., Rosenberg M. (2008). Nitrous oxide and the inhalation anesthetics. Anesth. Prog..

[73] Jones R. (1990). Desflurane and sevoflurane: Inhalation anaesthetics for this decade?. Br. J. Anaesth..

[74] Khan K.S., Hayes I., Buggy D.J. (2014). Pharmacology of anaesthetic agents II: Inhalation anaesthetic agents. Contin. Educ. Anaesth. Crit. Care Pain.

[75] Zhou J.-X., Liu J. (2001). The effect of temperature on solubility of volatile anesthetics in human tissues. Anesth. Analg..

[76] Novak R. Anesthesia Facts for Non-Medical People: How Does the Anesthesiologist Decide What Dose of Anesthetic to Give a Patient?. https://theanesthesiaconsultant.com/2013/10/03/anesthesia-facts-for-non-medical-people-how-does-the-anesthesiologist-decide-what-dose-of-anesthetic-to-give-a-patient/.

[77] Pace N.L., Stylianou M.P., Warltier D.C. (2007). Advances in and limitations of up-and-down methodology: A precis of clinical use, study design, and dose estimation in anesthesia research. Anesthesiology.

[78] Zezo D. Anesthesia Key. https://aneskey.com/inhalation-anesthetics-2/.

[79] Vernice N.A., Meydan C., Afshinnekoo E., Mason C.E. (2020). Long-term spaceflight and the cardiovascular system. Precis. Clin. Med..

[80] Gallo C., Ridolfi L., Scarsoglio S. (2020). Cardiovascular deconditioning during long-term spaceflight through multiscale modeling. Npj Microgravity.

[81] Tortorici M.A., Kochanek P.M., Poloyac S.M. (2007). Effects of hypothermia on drug disposition, metabolism, and response: A focus of hypothermia-mediated alterations on the cytochrome P450 enzyme system. Crit. Care Med..

[82] Julian Stine W.F. (2013). Anaesthesia at a Glance.

[83] Edgington T.L., Muco E., Maani C.V. (2021). Sevoflurane. StatPearls [Internet].

[84] Brioni J.D., Varughese S., Ahmed R., Bein B. (2017). A clinical review of inhalation anesthesia with sevoflurane: From early research to emerging topics. J. Anesth..

[85] Eis S., Kramer J. (2021). Anesthesia inhalation agents cardiovascular effects. StatPearls [Internet].

[86] Sonkajärvi E., Rytky S., Alahuhta S., Suominen K., Kumpulainen T., Ohtonen P., Karvonen E., Jäntti V. (2018). Epileptiform and periodic EEG activities induced by rapid sevoflurane anaesthesia induction. Clin. Neurophysiol..

[87] Sio L.C.L.O., dela Cruz R.G.C., Bautista A.F. (2017). Sevoflurane and renal function: A meta-analysis of randomized trials. Med. Gas Res..

[88] Wang C.-m., Chen W.-c., Zhang Y., Lin S., He H.-f. (2021). Update on the mechanism and treatment of sevoflurane-induced postoperative cognitive dysfunction. Front. Aging Neurosci..

[89] Patel S.S., Goa K.L. (1995). Desflurane. Drugs.

[90] Nishikawa K., Harrison N.L. (2003). The actions of sevoflurane and desflurane on the γ-aminobutyric acid receptor type A: Effects of TM2 mutations in the α and β subunits. J. Am. Soc. Anesthesiol..

[91] Khan J., Liu M. (2021). Desflurane. StatPearls [Internet].

[92] Kong C., Chew S., Ip-Yam P. (2000). Intravenous opioids reduce airway irritation during induction of anaesthesia with desflurane in adults. Br. J. Anaesth..

[93] NYSORA Inhaled Anesthetics. https://www.nysora.com/anesthesia/inhaled-anesthetics/.

[94] Weiskopf R.B., Cahalan M.K., Eger E.I., Yasuda N., Rampil I.J., Ionescu P., Lockhart S.H., Johnson B.H., Freire B., Kelley S. (1991). Cardiovascular actions of desflurane in normocarbic volunteers. Anesth. Analg..

[95] Franks N.P., Dickinson R., de Sousa S.L., Hall A.C., Lieb W.R. (1998). How does xenon produce anaesthesia?. Nature.

[96] Marx T., Schmidt M., Schirmer U., Reinelt H. (2000). Xenon anaesthesia. J. R. Soc. Med..

[97] Nakata Y., Goto T., Morita S. (1997). Comparison of inhalation inductions with xenon and sevoflurane. Acta Anaesthesiol. Scand..

[98] Goto T., Saito H., Shinkai M., Nakata Y., Ichinose F., Morita S. (1997). Xenon provides faster emergence from anesthesia than does nitrous oxide-sevoflurane or nitrous oxide-isoflurane. J. Am. Soc. Anesthesiol..

[99] Rossaint R., Reyle-Hahn M., Schulte am Esch J., Scholz J., Scherpereel P., Vallet B., Giunta F., Del Turco M., Erdmann W., Tenbrinck R. (2003). Multicenter randomized comparison of the efficacy and safety of xenon and isoflurane in patients undergoing elective surgery. J. Am. Soc. Anesthesiol..

[100] Chakkarapani E., Thoresen M., Liu X., Walloe L., Dingley J. (2012). Xenon offers stable haemodynamics independent of induced hypothermia after hypoxia–ischaemia in newborn pigs. Intensiv. Care Med..

[101] Eger E.I. (2004). Characteristics of anesthetic agents used for induction and maintenance of general anesthesia. Am. J. Health Syst. Pharm..

[102] Hosseinzadeh H., Eidy M., Golzari S.E., Vasebi M. (2013). Hemodynamic stability during induction of anesthesia in elderlyPatients: Propofol+ ketamine versus propofol+ etomidate. J. Cardiovasc. Thorac. Res..

[103] White P.F., Way W.L., Trevor A.J. (1982). Ketamine—Its pharmacology and therapeutic uses. J. Am. Soc. Anesthesiol..

[104] Reade M.C., Finfer S. (2014). Sedation and delirium in the intensive care unit. N. Engl. J. Med..

[105] Talke P., Tayefeh F., Sessler D.I., Jeffrey R., Noursalehi M., Richardson C. (1997). Dexmedetomidine does not alter the sweating threshold, but comparably and linearly decreases the vasoconstriction and shivering thresholds. J. Am. Soc. Anesthesiol..

[106] Callaway C.W., Elmer J., Guyette F.X., Molyneaux B.J., Anderson K.B., Empey P.E., Gerstel S.J., Holquist K., Repine M.J., Rittenberger J.C. (2015). Dexmedetomidine reduces shivering during mild hypothermia in waking subjects. PLoS ONE.

[107] Seo H.-y., Oh B.-j., Park E.-j., Min Y.-g., Choi S.-c. (2015). Dexmedetomidine Use in Patients with 33oC Targeted Temperature Management: Focus on Bradycardia as an Adverse Effect. Korean J. Crit. Care Med..

[108] Roy R.C. (2025). Agents That Came in From the Cold: Enflurane, Isoflurane, Desflurane, and Sevoflurane. Anesth Analg..

[109] De Wit F., Van Vliet A., De Wilde R., Jansen J., Vuyk J., Aarts L., De Jonge E., Veelo D., Geerts B. (2016). The effect of propofol on haemodynamics: Cardiac output, venous return, mean systemic filling pressure, and vascular resistances. Br. J. Anaesth..

[110] Folino T.B., Muco E., Safadi A.O., Parks L.J. (2022). Propofol. StatPearls [Internet].

[111] Perkins G.D., Gräsner J.-T., Semeraro F., Olasveengen T., Soar J., Lott C., Van de Voorde P., Madar J., Zideman D., Mentzelopoulos S. (2021). European resuscitation council guidelines 2021: Executive summary. Resuscitation.

[112] Hawkins W.A., Kim J.Y., Smith S.E., Newsome A.S., Hall R.G. (2022). Effects of propofol on hemodynamic profile in adults receiving targeted temperature management. Hosp. Pharm..

[113] Thomsen J.H., Nielsen N., Hassager C., Wanscher M., Pehrson S., Køber L., Bro-Jeppesen J., Søholm H., Winther-Jensen M., Pellis T. (2016). Bradycardia during targeted temperature management: An early marker of lower mortality and favorable neurologic outcome in comatose out-of-hospital cardiac arrest patients. Crit. Care Med..

[114] Smischney N.J., Nicholson W.T., Brown D.R., Gallo De Moraes A., Hoskote S.S., Pickering B., Oeckler R.A., Iyer V.N., Gajic O., Schroeder D.R. (2019). Ketamine/propofol admixture vs etomidate for intubation in the critically ill: KEEP PACE Randomized clinical trial. J. Trauma Acute Care Surg.

[115] Charuvi S., Sahajananda H., Dwajani S. (2020). Suppression of Cortisol Levels by a Bolus Dose of Etomidate in Patients Undergoing Laparoscopic Cholecystectomy. J. Med. Sci..

[116] Gallanosa A., Stevens J.B., Hendrix J.M., Quick J. (2025). Glycopyrrolate.

[117] Wolf N., Pendergrass W., Singh N., Swisshelm K., Schwartz J. (2008). Radiation cataracts: Mechanisms involved in their long delayed occurrence but then rapid progression. Mol. Vis..

[118] Chodick G., Bekiroglu N., Hauptmann M., Alexander B.H., Freedman D.M., Doody M.M., Cheung L.C., Simon S.L., Weinstock R.M., Bouville A. (2008). Risk of cataract after exposure to low doses of ionizing radiation: A 20-year prospective cohort study among US radiologic technologists. Am. J. Epidemiol..

[119] Ainsbury E.A., Barnard S., Bright S., Dalke C., Jarrin M., Kunze S., Tanner R., Dynlacht J.R., Quinlan R.A., Graw J. (2016). Ionizing radiation induced cataracts: Recent biological and mechanistic developments and perspectives for future research. Mutat. Res. Rev. Mutat. Res..

[120] Rastegar Z., Eckart P., Mertz M. (2002). Radiation-induced cataract in astronauts and cosmonauts. Graefe’s Arch. Clin. Exp. Ophthalmol..

[121] Zhang X., Liu B., Lal K., Liu H., Tran M., Zhou M., Ezugwu C., Gao X., Dang T., Au M.L. (2023). Antioxidant System and Endoplasmic Reticulum Stress in Cataracts. Cell. Mol. Neurobiol..

[122] Kulbay M., Wu K.Y., Nirwal G.K., Bélanger P., Tran S.D. (2024). Oxidative Stress and Cataract Formation: Evaluating the Efficacy of Antioxidant Therapies. Biomolecules.

[123] Li J., Buonfiglio F., Zeng Y., Pfeiffer N., Gericke A. (2024). Oxidative Stress in Cataract Formation: Is There a Treatment Approach on the Horizon?. Antioxidants.

[124] Klein A., Meek T., Allcock E., Cook T., Mincher N., Morris C., Nimmo A., Pandit J., Pawa A., Rodney G. (2021). Recommendations for standards of monitoring during anaesthesia and recovery 2021: Guideline from the Association of Anaesthetists. Anaesthesia.

[125] Omairi A.M., Pandey S. (2023). Targeted Temperature Management (TTM, Therapeutic Hypothermia). StatPearls [Internet].

[126] Mathur S., Patel J., Goldstein S., Jain A. (2021). Bispectral Index. StatPearls [Internet].

[127] Fang Z.X., Eger E.I., Laster M.J., Chortkoff B.S., Kandel L., Ionescu P. (1995). Carbon monoxide production from degradation of desflurane, enflurane, isoflurane, halothane, and sevoflurane by soda lime and Baralyme. Anesth. Analg..

[128] Żalikowska-Gardocka M., Przybyłkowski A. (2020). Review of parenteral nutrition-associated liver disease. Clin. Exp. Hepatol..

[129] Polderman K.H., Herold I. (2009). Therapeutic hypothermia and controlled normothermia in the intensive care unit: Practical considerations, side effects, and cooling methods. Crit. Care Med..

[130] Nordfjeld K., Rasmussen M., Jensen V.G. (1983). Storage of mixtures for total parenteral nutrition--long-term stability of a total parenteral nutrition mixture. J. Clin. Hosp. Pharm..

[131] Cahill T., Cope H., Bass J.J., Overbey E.G., Gilbert R., da Silveira W.A., Paul A.M., Mishra T., Herranz R., Reinsch S.S. (2021). Mammalian and Invertebrate Models as Complementary Tools for Gaining Mechanistic Insight on Muscle Responses to Spaceflight. Int. J. Mol. Sci..

[132] Smith G., D’Cruz J.R., Rondeau B., Goldman J. (2024). General Anesthesia for Surgeons. StatPearls.

[133] Flores M.M., Singh B. (2023). Neuromuscular blocking agents. Anesthesia and Analgesia in Laboratory Animals.

[134] Huang L., Li M., Deng C., Qiu J., Wang K., Chang M., Zhou S., Gu Y., Shen Y., Wang W. (2022). Potential therapeutic strategies for skeletal muscle atrophy. Antioxidants.

[135] Jung K.T., Kim S.H., Lee H.Y., Dal Jung J., Yu B.S., Lim K.J., So K.Y., Lee J.Y., An T.H. (2014). Effect on thermoregulatory responses in patients undergoing a tympanoplasty in accordance to the anesthetic techniques during PEEP: A comparison between inhalation anesthesia with desflurane and TIVA. Korean J. Anesthesiol..

[136] Lahvic N., Liu M. (2021). Waste gas scavenging system. StatPearls [Internet].

[137] Bernard S.A., Smith K., Finn J., Hein C., Grantham H., Bray J.E., Deasy C., Stephenson M., Williams T.A., Straney L.D. (2016). Induction of therapeutic hypothermia during out-of-hospital cardiac arrest using a rapid infusion of cold saline: The RINSE trial (rapid infusion of cold normal saline). Circulation.

[138] Rosenberg H., Sambuughin N., Riazi S., Dirksen R. (2020). Malignant hyperthermia susceptibility. GeneReviews^®^ [Internet].

[139] Badjatia N. (2006). Therapeutic temperature modulation in neurocritical care. Curr. Neurol. Neurosci. Rep..

[140] Rhodes J.K.J., Sinclair H.L., Battison C.G., Harris B., Andrews P.J.D. (2015). Shivering management during therapeutic hypothermia in patients with traumatic brain injury: Protocol from the Eurotherm3235 trial. BMC Emerg. Med..

[141] Jain A., Gray M., Slisz S., Haymore J., Badjatia N., Kulstad E. (2018). Shivering treatments for targeted temperature management: A review. J. Neurosci. Nurs..

[142] Renew J.R., Ratzlaff R., Hernandez-Torres V., Brull S.J., Prielipp R.C. (2020). Neuromuscular blockade management in the critically Ill patient. J. Intensiv. Care.

